# Clinical and Imaging Data-Based Model for Predicting Reversible Posterior Leukoencephalopathy Syndrome (RPLS) in Pregnant Women With Severe Preeclampsia or Eclampsia and Analysis of Perinatal Outcomes

**DOI:** 10.1155/2022/6990974

**Published:** 2022-04-15

**Authors:** Peng An, Junyan Zhang, Yang Li, Peng Duan, Yan Hu, Xiumei Li, Zhongqiu Wang

**Affiliations:** ^1^Department of Radiology, The Affiliated Hospital of Nanjing University of Chinese Medicine, Jiangsu Province Hospital of Chinese Medicine, The First Clinical Medical College, 155 Hanzhong Road, Nanjing 210029, Jiangsu, China; ^2^Department of Radiology, Xiangyang No. 1 People's Hospital, Hubei University of Medicine, Xiangyang 441000, China; ^3^Department of Pharmacy and Laboratory, Xiangyang No. 1 People's Hospital, Hubei University of Medicine, Xiangyang 441000, China; ^4^Department of Obstetrics and Gynecology, Xiangyang No. 1 People's Hospital, Hubei University of Medicine, Xiangyang 441000, China; ^5^Department of Internal Medicine, Xiangyang No. 1 People's Hospital, Hubei University of Medicine, Xiangyang 441000, China

## Abstract

**Objective:**

This study aimed to investigate the risk factors of reversible posterior leukoencephalopathy syndrome (RPLS) in pregnant women with severe preeclampsia or eclampsia (SPE/E) based on a predicting model and to analyze the perinatal outcomes.

**Methods:**

From January 2015 to March 2020, 78 pregnant women data diagnosed with severe preeclampsia or eclampsia with cranial magnetic resonance imaging (MRI) and transcranial Doppler (TCD) screening in Xiangyang No. 1 People's Hospital and Jiangsu Province Hospital of Chinese Medicine were analyzed retrospectively. They were divided into the RPLS group (*n* = 33) and non-RPLS group (*n* = 45) based on the MRI results. The general clinical data (blood pressure, BMI, symptoms, and so forth), laboratory examination, TCD results, and perinatal outcomes in the two groups were compared. The risk factors of severe preeclampsia or eclampsia complicated with RPLS were analyzed by multivariate logistic regression. The prediction model and decision curve (DCA) were established according to the clinical-imaging data.

**Results:**

The univariate analysis showed that poor placental perfusion, hypertension emergency, use of two or more oral antihypertensive drugs, headache, white blood cell (WBC) count, platelet (PLT) count, lactate dehydrogenase (LDH), alanine aminotransferase (ALT), uric acid (UA), serum albumin (ALB), average flow velocity, and resistance index of the posterior cerebral and basilar arteries were significantly different in the RPLS group compared with the non-RPLS group (all *P* < 0.05). The multivariate logistic regression analysis showed that hypertensive emergency, headache, WBC, PLT, ALT, and average flow velocity of the basilar artery (BAAFV) were the risk factors in the RPLS group. The aforementioned clinical-imaging data modeling (general data model, laboratory examination model, TCD model, and combined model) showed that the combined model predicted RPLS better. DCA also confirmed that the net benefit of the combined model was higher. In addition, the incidence of postpartum hemorrhage, stillbirth, and preterm infants was higher in the RPLS group than in the non-RPLS group (all *P* < 0.05).

**Conclusions:**

More postpartum complications were detected in pregnant women with severe preeclampsia or eclampsia complicated with RPLS. Hypertensive emergency, headache, WBC, PLT, ALT, and BAAFV were the important risk factors for RPLS. The combined model had a better effect in predicting RPLS.

## 1. Introduction

Reversible posterior leukoencephalopathy syndrome (RPLS) is a clinical neuroimaging syndrome. Hinkey et al. first reported and proposed it in 1996. The occurrence of RPLS mainly depends on capillary filtration pressure and integrity of the blood-brain barrier. The clinical symptoms of RPLS mainly include neurological impairment, such as headache, epileptic attack, blurred vision, and mental or consciousness disorders [1, 2]. At present, the incidence rate of RPLS is unknown, the pathogenesis is not clear, and the clinical symptoms are not specific. However, pregnant women with eclampsia are the high-risk group of RPLS, with rapid onset and progress, easily endangering the lives of mothers and fetuses. Eclampsia is a clinical syndrome leading to multiple-organ function injury, with an incidence of about 3%–8% in pregnancy. More than 70% of preeclampsia (PE)-related deaths are related to nervous system complications, such as cerebral edema and intracerebral hemorrhage [3–5]. Previous studies have shown that postpartum brain injury persists in patients with brain edema, stroke, or hypoxic-white matter lesions during pregnancy, and the risk of cerebrovascular disease and cognitive impairment increases in the long term [6, 7]. The course of RPLS disease is usually reversible, and the prognosis is good based on medical intervention. The occurrence of these long-term complications can be slowed down through early detection of the distribution of RPLS lesions as well as active lifestyle intervention and blood pressure management [6]. Magnetic resonance imaging (MRI) is the gold standard for analyzing preeclampsia complicated with nervous system injury, but direct clinical evidence for any specific symptom or indication for clinical significance is currently lacking [6, 8]. In other words, we cannot arbitrarily require every pregnant woman with preeclampsia to undergo a cranial MRI. The latest research of our group included general clinical data, laboratory examination data, and transcranial Doppler (TCD) data for comprehensive analysis and achieved good prediction benefits. The clinical data of 78 patients with severe preeclampsia or eclampsia (SPE/E) who underwent cranial MRI and TCD were analyzed retrospectively. The risk factors of RPLS were analyzed, and a prediction model was established to provide a basis for the early identification of SPE/E with RPLS.

## 2. Methods

### 2.1. General Data

Pregnant women with SPE/E delivered in Xiangyang No. 1 People's Hospital and Jiangsu Province Hospital of Chinese Medicine from January 2015 to March 2020 were retrospectively enrolled and followed up until September 5, 2021. Their blood pressure and symptoms at admission, laboratory test results, and so on were recorded. The inclusion criterion was pregnant women diagnosed with SPE/E undergoing cranial MRI and TCD screening during hospitalization. The exclusion criterion was pregnant women with previous serious neurological or internal diseases affecting or shortening life expectancy. A total of 78 pregnant women with SPE/E were included in the study. They were divided into RPLS group (*n* = 33) and non-RPLS group (*n* = 45) based on cranial MRI results.

### 2.2. Diagnostic Criteria

Eclampsia: Defined as convulsions occurring in pregnant women with preeclampsia that cannot be explained by other reasons.

Severe preeclampsia: Defined as any of the following adverse conditions: systolic blood pressure ≥160 mm Hg (1 mm Hg = 0.133 kPa) or diastolic blood pressure ≥110 mm Hg; 24-h urinary protein ≥5 g or random urinary protein ≥ (+++); abnormal renal function; persistent headache or visual impairment or other neurological symptoms; pulmonary edema and heart failure; upper abdominal or right upper abdominal pain; hypoproteinemia with effusion; impaired liver function; abnormality of blood and blood-forming tissues; fetal growth restriction, and so forth.

Classification of hypertension: The blood pressure collected in this study was the average blood pressure of pregnant women in the hospital (rather than the highest blood pressure during hospitalization) to avoid the influence of antihypertensive drugs. (1) Grade 1 hypertension was defined as systolic blood pressure of 140–159 mm Hg and/or diastolic blood pressure of 90–99 mm Hg; (2) Grade 2 hypertension was defined as systolic blood pressure of 160–179 mm Hg and/or diastolic blood pressure of 100–109 mm Hg; (3) Grade 3 hypertension was defined as systolic blood pressure of ≥180 mm Hg and/or diastolic blood pressure of ≥110 mm Hg.

If an acute attack lasted more than 15 min, it was persistent severe hypertension, which was an emergency of hypertension. Those who used two or more kinds of antihypertensive drugs referred to those who could not achieve the target blood pressure with single antihypertensive drug treatment and needed two or more kinds of oral antihypertensive drugs or even intravenous or intravenous-equivalent drugs. Fetal growth restriction meant that the estimated fetal weight (EFW) was less than the 10th percentile of the corresponding gestational age weight [7, 9, 10].

### 2.3. MRI and TCD Examination Methods

MRI: Participants had a baseline or above the MRI brain examination in 2015–2020 and returned for a repeat MRI examination after 3–6, 6–12, and 12–18 months. MRI screening routinely included conventional MRI sequence T1-weighted imaging (T1WI) + T2-weighted imaging (T2WI) + fluid-attenuated inversion recovery sequence (FLAIR). For patients with headaches and other special symptoms, MRI screening included diffusion-weighted imaging (DWI) + MR venography (MRV) sequence, or other sequences were added based on the personal condition so as to distinguish them from pregnancy-related encephalopathy such as cerebral venous sinus thrombosis. The imaging diagnosis results were summarized and confirmed by two imaging physicians after reading the films. In case of disagreement, the third senior imaging physician was invited to make a joint and key diagnosis. A 3.0 T MRI scanner (Siemens AG Magnetom Prism, Germany) and a 1.5-T MRI scanner (Philips Achieva, the Netherlands) were used for MRI examination. The technical parameters were as follows: (1) T1WI: repetition time (TR) 2000 ms, echo time (TE) 20 ms, and number of signal averaged (NSA) = 2; T2WI:TR 2139 ms, TE 80 ms, and NSA = 3; (2) FLAIR: TR 7000 ms, TE 157 ms, NSA = 2, time reversal (TI) 2200 ms, FOV 240 × 240 mm, layer thickness 6 mm, and spacing 1 mm. (3) DWI: TR 2308 ms, TE 88 ms, number of layers = 18, layer thickness 6 mm, and NSA = 2; (4) MRV: TR 18 ms, TE 88 ms, flip angle 10°, FOV 230 × 120 × 178 mm, number of layers = 150, matrix 232 × 137, and NSA = I [8].

TCD: The desktop transcranial Doppler ultrasound diagnostic instrument of DWL Company in Germany was adopted, and the probe frequency range was 2–2.5 MHz. The probe detected anterior circulation and posterior circulation from the temporal window, occipital window, and eye window. The detection indexes of anterior circulation included middle cerebral artery (MCA), anterior cerebral artery (ACA), and terminal end of the internal carotid artery (TICA) and posterior cerebral artery (PCA). The detection indexes of posterior circulation included the basilar artery (BA) and vertebral artery (VA).

### 2.4. Statistical Method

Spss 22.0 software (IBM, Armonk, NY, USA) and *R* software (R foundation for statistical computing, version 3.4.1; https://www.r-project.org/) were used for statistical analysis. The measurement data are expressed mean ± SD. The independent sample *t*-test is used for the comparison between the data groups that conform to the normal distribution, whereas the rank sum test is used for those that do not conform to the normal distribution. Counting data were expressed by frequency or percentage, and X2 test or corrected x2 test was used for intergroup comparison. The OR value and 95% CI of risk factors were calculated by multivariate logistic regression analysis. The difference was statistically significant (*P* < 0.05). Then, the receiver operating characteristic (ROC) curve was generated, and the area under the curve (AUC) was used to evaluate the accuracy of general data model, laboratory examination model, TCD model, and combined model in predicting RPLS. The higher the AUC and *P* value < 0.05 (two tailed), the higher the prediction accuracy, which shows statistical significance, and then conduct decision curve analysis (DCA). The clinical usefulness was determined by quantifying the net benefits under different threshold probabilities in the model [9, 11].

## 3. Results

### 3.1. General Information

**Figure 1:**
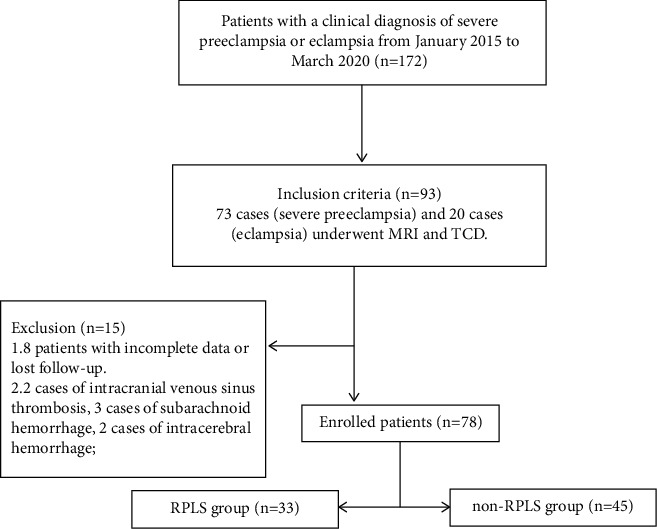
Flow chart showing inclusion and exclusion of subjects (SPE/E) in this study.

From January 2015 to March 2020, 172 cases of severe preeclampsia or eclampsia delivered naturally or by a cesarean section in our hospital, and 73 cases (severe preeclampsia) and 20 cases (eclampsia) underwent MRI and TCD, respectively. Eight patients with incomplete data or lost to follow-up were excluded. Furthermore, 85 patients, comprising 33 patients with RPLS, 2 patients with intracranial venous sinus thrombosis, 3 patients with subarachnoid hemorrhage, 2 patients with intracerebral hemorrhage, and 45 normal patients, were included. No significant difference was found between the two groups in terms of age, gestational weeks, assisted reproduction, multiple pregnancies, immune system diseases, and history of chronic hypertension (*P* > 0.05). The univariate analysis showed that poor placental perfusion, hypertension emergency, use of two or more kinds of oral antihypertensive drugs, and headache were significantly different in pregnant women with RPLS compared with those without RPLS (*P* < 0.05) (Figure 1 and Table 1).

### 3.2. Laboratory Test Results

No significant difference was found in red blood cell (RBC) count, hemoglobin (HB), activated partial thromboplastin time (APTT), 24-h urinary protein quantification, aspartate aminotransferase (AST), serum total cholesterol (TC), urea nitrogen, and creatinine levels between pregnant women with RPLS and those without RPLS (All *P* > 0.05). The WBC count; PLT count; and levels of LDH, ALT, UA, and serum albumin were compared between the two groups, and the differences were statistically significant (all *P* < 0.05) (Table 2).

### 3.3. TCD Examination Results

The blood flow resistance and average flow velocity of the posterior cerebral artery and basilar artery in pregnant women with RPLS were significantly different from those in pregnant women without RPLS (all *P* < 0.05). No significant difference was observed in blood flow parameters of ACA, MCA, carotid artery, and vertebral artery between the two groups (all *P* > 0.05) (Table 3).

### 3.4. Analysis of Predictive Factors of Cranial MR Abnormalities

**Figure 2:**
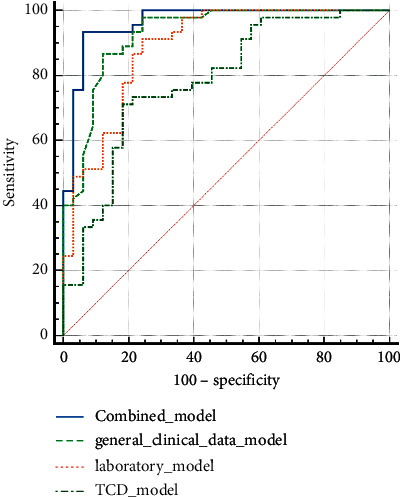
Among the four prediction models based on the above risk factors, the combined model has good prediction effect, and the area under the curve is the largest.

**Figure 3:**
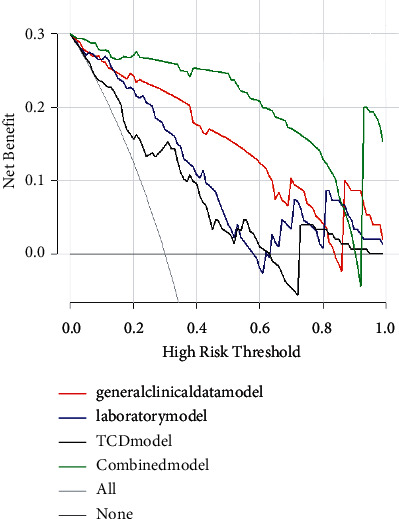
The decision curve also confirms that the net benefit of the combined model is high.

The aforementioned risk factors were analyzed by binary logistic regression. Hypertensive emergency, headache, WBC, PLT, ALT, and BAAFV were independent risk factors for severe preeclampsia or eclampsia complicated with RPLS. Four RPLS risk factor models (general clinical data model, laboratory inspection model, TCD model, and combined model) were established. The prediction value of the combined model was found to be high, and the combined model was the best decision to maximize the net benefit compared with the other three models (Figures 2, 3 and Table 4).

### 3.5. 3- to 18-Month Postpartum Follow-Up of RPLS in Pregnant Women

Among 33 pregnant women with RPLS, 31 completed telephone follow-ups. The follow-up rate was 93.93%, and the median follow-up time was 8 months (3–18 months). All follow-up patients survived without residual neurological sequelae, such as behavior, movement, and vision and language disorders. Eight cases still complained of intermittent headaches, especially when the blood pressure was poorly controlled. During the follow-up, five patients were transferred to neurology consultation and treatment, including short-term antiepileptic, anticoagulant, nerve nutrition, and other treatment measures. In 30 patients with RPLS, the cerebral cortical edema was alleviated or disappeared after 6–12 weeks postpartum.

## 4. Discussion

### 4.1. Mechanism of Cerebrovascular Injury in Preeclampsia

At present, the mechanism of cerebrovascular injury in preeclampsia is unknown. Mostly, cerebrovascular injury is related to the increase in blood-brain barrier permeability caused by the dysfunction of vascular endothelial cells and the abnormal ability of cerebrovascular self-regulation. Brain hyperperfusion and vasospasm are the most crucial research mechanisms. The common imaging manifestations of nervous system injury related to preeclampsia are stroke and RPLS [12, 13].

### 4.2. Prediction and Analysis of RPLS in Severe Preeclampsia or Eclampsia

In this study, no significant difference was found in age, gestational weeks of delivery, assisted reproduction, multiple pregnancies, immune system diseases, history of chronic hypertension, RBC count, HB, APTT, and 24-h urinary protein, AST, TC, urea nitrogen, and creatinine levels between the RPLS and non-RPLS groups. It was suggested that the aforementioned factors were not risk factors for severe preeclampsia or eclampsia complicated with RPLS.

**Figure 4:**
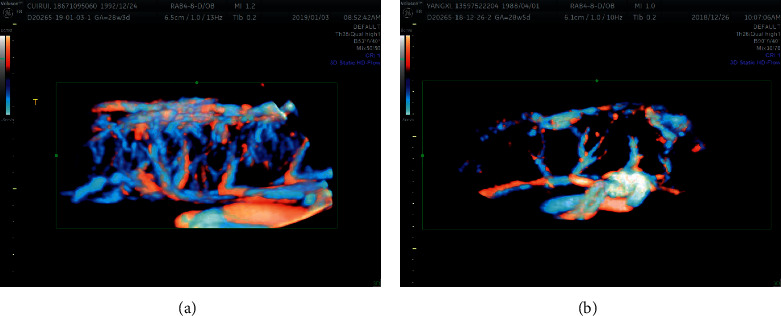
The application of 3D-HD Flow technology in the evaluation of placental vascular perfusion; the placental vascular perfusion in the non-RPLS group was higher than that in the RPLS group. (a) non-RPLS, (b) RPLS.

1. Acute or subacute neurological symptoms (headache, epileptiform seizures, blurred vision, consciousness or mental disorders, including nausea, vomiting, focal neurological deficit, and other symptoms) are the most intuitive manifestations of preeclampsia complicated with neurological injury, which need to be evaluated by MRI actively. In preeclampsia cases with neurological symptoms, the incidence of abnormal cranial MRI was 55.3%. Headache is a high-risk factor for RPLS and a precursor of eclampsia. Cortical blindness is also a unique manifestation of RPLS [11, 14, 15]. This study found that in preeclampsia, neurological symptoms such as headache (*P* < 0.05) was independent risk factors for RPLS, but the blurred vision is also a potential factor that cannot be ignored (The *p* value 0.072 is close to 0.05). Therefore, pregnant women should be vigilant once they have the aforementioned symptoms. The convulsive symptoms were nonspecific. No significant difference was noted between the two groups in this study (*P* > 0.05).
2. In the course of SPE/E, hypertension emergency and use of two or more antihypertensive drugs can increase the risk of RPLS. Cerebral blood vessels are early target organs affected by hypertension. In the case of sustained hypertension emergency, the risk of serious adverse events such as hypertensive encephalopathy, intracerebral hemorrhage, cerebral infarction, and RPLS increases, and long-term high blood pressure levels lead to cerebral arteriosclerosis and hypoxic changes in brain tissue, which is related to the decline in cognitive function and the occurrence of dementia after many years [7, 11, 16]. This is consistent with the Chinese proverb “Obese pregnant women will be stupid for at least three years.” A retrospective study analyzed the data of patients with preeclampsia or eclampsia-related stroke and RPLS. It also put forward the view that we should pay close attention to maternal hypertension, so hypertensive emergency monitoring is more crucial [6, 9].
3. Soma Pillay et al. found that patients with preeclampsia who needed to use an antihypertensive drug had a significantly increased risk of white matter lesions during pregnancy and 6-month postpartum. Kurosaki et al. found that the diastolic blood pressure of patients with preeclampsia negatively correlated with the function of cognitive nerve units in the brain. These results suggested that hypertension had an important impact on cerebrovascular injury. Active antihypertensive treatment can avoid stroke and other complications caused by severe hypertension and alleviate the development course of white matter lesions. However, antihypertensive treatment did not significantly reduce cerebral perfusion volume in patients with preeclampsia. As a risk factor for RPLS, the use of two or more antihypertensive drugs also objectively confirmed the impact of uncontrollable hypertension on cerebrovascular injury. In other words, although the combined use of antihypertensive drugs reduced the incidence of stroke, RPLS did not improve [17–20].
4. Endothelial dysfunction caused by abnormal placental vascular perfusion in eclampsia may be related to abnormal cerebrovascular function. The same results were also observed in the placental blood flow perfusion in patients with RPLS using prenatal ultrasound 3D-flow function analysis, and the postpartum placental vascular cast was confirmed (Figure 4). Similarly, fetal growth restriction caused by poor placental perfusion was not a direct factor leading to cerebrovascular injury, but this clinical manifestation suggested that patients had placental vascular dysplasia and endothelial injury, and the risk of RPLS was increased [14, 21].
5. When eclampsia is complicated with RPLS, pregnant women with increased cerebral blood flow, hyperperfusion, and increased blood-brain barrier permeability need to undergo MRI. However, we cannot force all pregnant women with eclampsia to undergo MRI due to its high cost and claustrophobic symptoms. Therefore, a more convenient TCD was chosen for cerebral blood flow assessment, and good benefits were achieved. This study found that the blood flow resistance of posterior cerebral artery and basilar artery decreased and the average flow velocity increased in pregnant women with RPLS, showing the sign of “low resistance and high output.” This novel important finding provides an opportunity for the universal screening of RPLS in the future.
6. Uric acid and lactate dehydrogenase levels are one of the indicators reflecting the severity of endothelial dysfunction, which have a high correlation with RPLS. This study found that both significantly increased in the RPLS group, suggesting that endothelial inflammatory injury might play an important role in the process of brain edema of RPLS, which could be used as a predictor of the degree of brain edema of RPLS [22–24].
7. This study found that the ALT level in the RPLS group was higher than that in the non-RPLS group, and the AST and ALT levels in the two groups were significantly higher than the normal value, indicating varying degrees of damage to the liver function of pregnant women with pregnancy-induced hypertension or SPE/E combined with RPLS, but this was not a specific index [24]. The coagulation function of pregnant women with SPE/E was analyzed through coagulation indexes and PLT. The results showed that pregnant women with SPE/E had obvious blood hypercoagulability and PLT consumption, which was of great significance for condition evaluation, monitoring, prevention, and treatment. PLT in the RPLS group was significantly lower than that in the non-RPLS group, and PLT was also one of the effective risk factors for RPLS [23, 25].
8. Albumin and 24-h urinary protein quantification are good indicators for observing preeclampsia, which are related to the risk of brain edema and ascites. The serum albumin levels in the RPLS group is lower than that in the non-RPLS group, which also showed that serum albumin levels could be used as a predictor of the degree of brain edema of RPLS. Although the involvement of 24-h urinary protein in the prediction and evaluation of RPLS has decreased, some studies suggested that Hb and 24-h urinary protein levels had significant changes in pregnant women with preeclampsia [24, 26]. In this study, no significant difference was found in the RBC count, Hb, and 24-h urinary protein level between the group with RPLS and the group without RPLS, indicating that RBC, Hb, and 24-h urinary protein were predictors and evaluation indexes of severe preeclampsia or eclampsia, but whether to predict the combination of RPLS needs to be further studied.
9. In this study, the WBC count of pregnant women in the RPLS group was significantly higher than that in the non-RPLS group, which might be related to the relatively serious condition and enhanced inflammation or stress response of pregnant women in the RPLS group.
10. It is reported that the main factor directly related to perinatal outcome is gestational age. However, this study found that the incidence of postpartum hemorrhage, preterm infants, and stillbirths was slightly higher in the combined RPLS group than in the non-RPLS group. No significant difference was found in placental abruption, neonatal asphyxia, hypoxic-ischemic encephalopathy (HIE), and neonatal respiratory distress syndrome (RDS) between the two groups. It was suggested that eclampsia combined with RPLS had a certain impact on maternal complications and perinatal infants [3, 4, 24] (Table 5).

**Figure 5:**
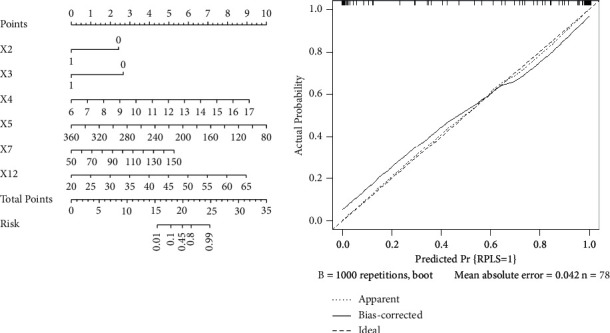
The nomogram based on the risk factors of the combined model has been highly praised by clinicians. Notes: X2 = hypertensive emergency; X3 = headache; X4 = headache; X5 = PLT; X7 = ALT; X12 = BAAFV; X4 = WBC.

In addition, four RPLS prediction models (general clinical data model, laboratory inspection model, TCD model, and combined model) were established in this study based on the aforementioned risk factors. The results suggested that the prediction value of the combined model was high. DCA also confirmed that the net benefit of the combined model was high. The nomogram tool based on the risk factors of the combined model simplified the prediction method of RPLS and has been applied in clinical practice (Figure 5). Our proposed algorithm is better than existing Carlos R's algorithm [16].First, this study was more comprehensive and systematic (including complete medical history, laboratory examination, hypertension emergency, etc) and had a higher sample size (33 vs. 17) than previous studies, and we established four models to compare the predicted effectiveness using DCA. Second, the newly added placental ultrasound perfusion and TCD parameters had important clinical prediction values, and nomograms have also achieved better performance, which were not reported earlier. Although the prognosis of SPE/E pregnant women with RPLS was better than that of women with RPLS caused by other causes, and the course of the disease was benign and reversible. However, timely diagnosis and correct and effective intervention are important. Therefore, early identification of RPLS is of great significance. The continuous development of RPLS caused by neglected high cerebral blood flow perfusion is bound to lead to white matter dysfunction, slow response/insensitivity, and even serious complications, such as stroke and intracerebral hemorrhage. Therefore, once the aforementioned early warning signs appear in pregnant women with SPE/E, MRI examination should be carried out immediately; timely diagnosis and early intervention can improve the prognosis [5, 9, 16, 27].

### 4.3. Limitations

This study included only the data of 93 pregnant women with SPE/E who underwent cranial MRI and TCD, accounting for 54.07% (93/172) in this group, of which about 35.48% (33/93) had RPLS. Considering that in clinical practice, only patients with severe preeclampsia have improved clinical systemic evaluation and imaging examination, the data of this study ignored most mild preeclampsia cases, which is biased and needs to be confirmed by further prospective multiinstitutional studies in the later stage. The difference in RPLS between eclampsia and severe preeclampsia could not be compared due to insufficient data, and hence, training and test sets could not be set up. Probably, the results slightly varied as per the diversity characteristics of the participants, but it was insignificant. Also, only the high-risk factors for RPLS were reported to help clinicians select the target population undergoing MRI examination. However, the impact of these MRI signs on pregnant women's long-term cognition and health status needs to be studied in the future.

## 5. Conclusions

In conclusion, Hypertensive emergency, headache, WBC, PLT, ALT, and BAAFV, and so forth are independent high-risk factors for RPLS. A clinical-imaging data-based combined model for predicting RPLS is promising and may achieve more clinical benefits.

## Figures and Tables

**Table 1:** General data of pregnant women in the two groups [*n* (%)] (general clinical data model).

Category	RPLS group	Non-RPLS group	Univariate analysis		Multivariate analysis	
	*(n* = 33)	*(n* = 45)	*X* ^2^ */t*	*P*	*P*	Hazard ratio
Age (years)	32.64 ± 9.41	33.83 ± 9.51	0.551	0.584		
Gestational week of delivery (weeks)	33.51 ± 3.12	33.69 ± 3.21	0.269	0.788		
Abortion history	21	30	0.077	0.781		
Primipara	16	26	0.661	0.416		
Assisted reproduction	11	16	0.041	0.838		
Prepregnancy BMI ≥30 kg/m^2^	24	27	1.362	0.243		
Multiple pregnancies	3	5	0.0844	0.771		
Diabetes history	5	7	0.0023	0.961		
Immune disorders	2	4	0.2144	0.643		
Poor placental perfusion	30	29	2.788	0.007^*∗*^	0.031^*∗*^	3.503 (1.121–10.948)
History of eclampsia	6	9	0.04	0.84		
Hypertensive emergency	23	13	3.855	*P* < 0.05^*∗*^	0.001^*∗*^	7.248 (2.306–22.784)
≥2 antihypertensive drugs	22	11	4.061	*P* < 0.05^*∗*^		
History of hypertension	20	26	0.062	0.801		
HELLP syndrome	3	1	0.704	0.401		
Smoking or drinking history	6	10	0.19	0.662		
Headache	24	13	4.197	*P* < 0.05^*∗*^	0.009^*∗*^	4.464 (1.460–13.654)
Blurred vision	20	18	3.235	0.072		
Convulsions	8	5	1.541	0.127		
Mental or consciousness disorders	14	11	2.826	0.092		
Severe preeclampsia/eclampsia	16	23	0.0525	0.8187		

**Table 2:** Laboratory test results of two groups (laboratory model).

Category	RPLS group	Non-RPLS group	Univariate analysis		Multivariate analysis	
	(*n* = 33)	(*n* = 45)	*X* ^2^ */t*	*P*	*P*	Hazard ratio
RBC (x10^12^/L)	4.16 ± 0.355	4.04 ± 0.315	1.55	0.124		
Hb (g/L)	113.31 ± 24.26	113.11 ± 19.43	0.038	0.97		
WBC (x10^9^/L)	12.21 ± 1.99	11.02 ± 2.22	2.415	0.018^*∗*^	0.011^*∗*^	0.641 (0.456–0.902)
PLT (x10^9^/L)	177.58 ± 43.81	215.12 ± 48.67	3.507	0.001^*∗*^	0.010^*∗*^	1.023 (1.005–1.040)
APTT (s)	31.97 ± 2.01	32.08 ± 2.36	0.227	0.821		
24 h urinary protein (g)	4.82 ± 1.69	5.37 ± 1.95	1.33	0.198		
LDH (U/L)	583.39 ± 112.68	525.34 ± 120.61	2.158	0.034^*∗*^	0.032^*∗*^	0.994 (0.989–1.000)
AST (U/L)	151.69 ± 38.61	138.71 ± 35.98	1.509	0.135		
ALT (U/L)	110.74 ± 22.21	97.74 ± 21.87	2.576	0.012^*∗*^	0.006^*∗*^	0.959 (0.930–0.988)
Uric acid (mmol/L)	558.12 ± 87.31	496.27 ± 72.82	3.406	0.001^*∗*^	0.039^*∗*^	0.992 (0.984–1.000)
TC (mmol/L)	5.73 ± 1.48	5.84 ± 1.28	0.347	0.73		
Urea nitrogen (mmol/L)	5.95 ± 1.59	5.98 ± 1.81	0.095	0.925		
Creatinine (*μ*mol/L)	65.94 ± 21.88	69.33 ± 16.91	0.772	0.442		
Serum albumin (g/L)	23.82 ± 3.29	25.88 ± 3.05	2.840	0.006^*∗*^	0.042^*∗*^	1.241 (1.008–1.525)

**Table 3:** TCD results of two groups (TCD model).

Category	RPLS group	Non-RPLS group	Univariate analysis		Multivariate analysis	
	(*n* = 33)	(*n* = 45)	*X* ^2^ */t*	*P*	*P*	Hazard ratio
MCA (cm/s)						
AFV	70.42 ± 12.83	73.37 ± 14.25	0.942	0.349		
RI	0.65 ± 0.17	0.67 ± 0.15	0.561	0.577		
ACA (cm/s)						
AFV	49.83 ± 13.24	57.89 ± 14.75	1.586	0.117		
RI	0.65 ± 0.14	0.64 ± 0.12	0.348	0.736		
PCA (cm/s)						
AFV	42.38 ± 10.57	35.65 ± 8.72	3.077	0.003^*∗*^	0.668	0.984 (0.915–1.059)
RI	0.58 ± 0.19	0.68 ± 0.19	2.042	0.045^*∗*^	0.088	1.028 (0.996–1.060)
BA (cm/s)						
AFV	45.24 ± 8.91	38.58 ± 9.86	3.056	0.003^*∗*^	0.002^*∗*^	0.890 (0.826–0.958)
RI	0.48 ± 0.12	0.54 ± 0.11	2.025	0.046^*∗*^	0.048^*∗*^	1.050 (1.000–1.102)

AFV: average flow velocity.

**Table 4:** Risk factors of RPLS in pregnant women with severe preeclampsia or eclampsia by multivariate logistic regression analysis based on TCD, laboratory examination, and general clinical data (Combined model). ^*∗*^*P* < 0.05.

Category	*β* value	Standard error	Wald *X*^2^ value	*P* value	OR value	95% CI
Poor placental perfusion	1.446	1.139	1.613	0.204	4.247	0.456–39.585
Hypertensive emergency	2.412	1.167	4.270	0.039^*∗*^	11.157	1.132–109.929
≥2 antihypertensive drugs	0.946	0.492	3.691	0.055	2.576	0.981–6.763
Headache	2.601	1.239	4.409	0.036^*∗*^	13.483	1.189–152.896
WBC	0.922	0.362	6.481	0.011^*∗*^	0.398	0.196–0.809
PLT	0.032	0.014	5.157	0.023^*∗*^	1.032	1.004–1.061
LDH	0.001	0.004	0.113	0.737	0.999	0.991–1.007
ALT	0.055	0.027	4.107	0.043^*∗*^	0.946	0.897–0.998
Uric acid	0.008	0.006	1.904	0.168	0.992	0.981–1.004
Serum albumin	0.105	0.183	0.327	0.567	1.110	0.776–1.588
PCAVM	0.043	0.063	0.464	0.496	0.958	0.846–1.084
PCARI	0.010	0.028	0.130	0.719	0.990	0.936–1.046
BAAFV	0.135	0.064	4.417	0.036^*∗*^	0.874	0.771–0.991
BARI	0.051	0.043	1.384	0.239	1.052	0.967–1.146

**Table 5:** Comparison of perinatal outcomes between the two groups [*n* (%)].

Category	RPLS group (*n* = 33)	Non-RPLS group (*n* = 45)	*X* ^2^ *t*	*P*
Placental abruption	1	2	0.075	0.783
Preterm infants	20	13	7.846	0.005^*∗*^
Neonatal asphyxia	3	2	0.129	0.718
HIE	1	1	0.251	0.615
RDS	2	3	0.129	0.718
Stillbirths	5	0	4.978	0.025^*∗*^
Postpartum hemorrhage	6	1	4.143	0.041^*∗*^

## Data Availability

All data generated or analyzed during this study are included in this published article.
